# A case of disseminated sporotrichosis

**DOI:** 10.4102/sajid.v39i1.648

**Published:** 2024-07-12

**Authors:** Ashleigh A.S. Lamont, Kurai Tsoka, Sadhna Kooverjee, Michelle Venter

**Affiliations:** 1 Department of Internal Medicine, Faculty of Health Sciences, University of the Witwatersrand, Johannesburg, South Africa; 2 Department of Internal Medicine, Faculty of Health Sciences, University of Cape Town, Cape Town, South Africa

**Keywords:** *Sporothrix schenckii*, cutaneous sporotrichosis, disseminated sporotrichosis, osteo-articular sporotrichosis, human immunodeficiency virus

## Abstract

*Sporothrix schenckii* is a thermally dimorphic fungus found in soil and in plant matter. Cutaneous sporotrichosis is the most common manifestation and disseminated sporotrichosis is the rarest. Delayed diagnoses are not uncommon. We report a case of disseminated sporotrichosis involving the skin, bones and joints, with subsequent joint destruction, tissue loss and permanent disability.

**Contribution:**

This case report highlights the debilitating potential of sporotrichosis and its often elusive nature regarding diagnosis.

## Introduction

Sporotrichosis is the disease caused by infection with a fungus belonging to the genus Sporothrix that commonly causes lymphocutaneous lesions.^1^ The most common species causing disease in humans is *Sporothrix schenckii*; other species including *Sporothrix brasiliensis, Sporothrix glabosa, Sporothrix pallida* and *Sporothrix mexicana* have also been described.^1^ Sporotrichosis is reported worldwide; however, it is more common in regions with tropical or subtropical climates.^1^ It is abundant within vegetation, soil or contaminated matter.^2^ Transmission to humans is usually associated with traumatic inoculation of organic matter harbouring the Sporothrix fungus.^1^

*Sporothrix schenckii* is ubiquitous in the soils of South Africa.^3^ Sporotrichosis was first found in the gold mines of South Africa in 1927,^4^ with the largest outbreak between 1938 and 1947 in the gold mines of the Witwatersrand area.^5^

The cutaneous form presents as a papule that may ulcerate and drain at the site of inoculation.^6^ Similar lesions may develop along lymphatic drainage from the inoculation site.^6^

Disseminated forms of sporotrichosis are rare.^7^ Involvement of the lung, pleura, sinuses, meninges, liver, heart, kidneys, eyes, bone and joints are described.^6,7^ People living with human immunodeficiency virus (HIV) infection, excessive alcohol consumption, diabetes mellitus, steroid treatment, haematological cancers and solid organ transplant recipients are groups with increased risk for developing disseminated disease.^8^ Treatment of disseminated sporotrichosis often requires a longer course of antifungals than cutaneous sporotrichosis.^7^

This case report discusses disseminated sporotrichosis in an immunocompromised host, presenting with characteristic disseminated skin lesions as well as rare osteolytic involvement of multiple joints of the hands. Our patient highlights the debilitating nature of disseminated sporotrichosis and the difficulties in its diagnosis. A high index of suspicion is needed for this forgotten pathogen, endemic to South Africa.

## Case presentation

The patient’s initial presentation to Chris Hani Baragwanath Academic Hospital was in 2019, with a diffuse pruritic skin rash. There was loss of tissue on the patient’s nasal alae, a swollen and painful left hand and inability to move multiple fingers on both hands. The rash began as an ulcerative lesion on the base of the patient’s left middle finger in 2015. Between 2015 and 2019, the patient underwent multiple clinic visits and treatments with no improvement. Samples were not taken for laboratory testing during this time. Subsequent dissemination of similar lesions, with eventual coverage of the entire body was observed.

The patient, previously employed as a gardener, was residing in Soweto, South Africa at the first presentation. A strong history of alcohol use and cigarette smoking was found. The patient had HIV, which was first diagnosed 21 years ago. His HIV viral load was 14 400 HIV ribonucleic acid (RNA) copies/mL, which indicated virological failure. The CD4 count was 77 cell/µL, which indicated severe immunosuppression. This was because of poor adherence. The patient was on second-line therapy consisting of tenofovir disoproxil fumarate, emtricitabine and lopinavir with ritonavir.

On clinical examination, hyperkeratotic, hyperpigmented plaques and papular lesions were seen predominantly on the hands, arms and face. There was tissue loss of the nasal alae. There was no palpable lymphadenopathy. The patient’s hands exhibited fixed flexion deformities of the ring and middle fingers and the thumb, at the distal interphalangeal joints.

Multiple unsuccessful skin punch biopsies were sent for microscopy and culture, including fungal cultures that were sent during the year 2019. Mycological cultures of a biopsy specimen eventually revealed *S. schenckii* in August 2019. After a delay of over 4 years, a diagnosis of cutaneous sporotrichosis was made. Subsequent skin punch biopsies confirmed an underlying fungal aetiology, with features in keeping with a deep fungal infection on histology. Periodic-acid Schiff and Grocott-Gomori’s Methenamine Silver staining highlighted fungal organisms both superficially and deeper within the hair follicle in 2019. Initially, the patient responded well to an oral course of itraconazole but unfortunately defaulted treatment and follow-up in April 2020 because of social reasons.

**FIGURE 1:**
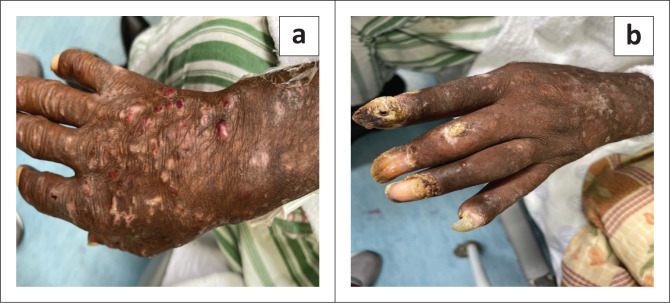
(a and b) Hyperkeratotic lesions on the dorsal aspect of the patient’s hand with fixed flexion deformities of the proximal and distal interphalangeal joints and hyperkeratinisation of the nails was noticed.

**FIGURE 2:**
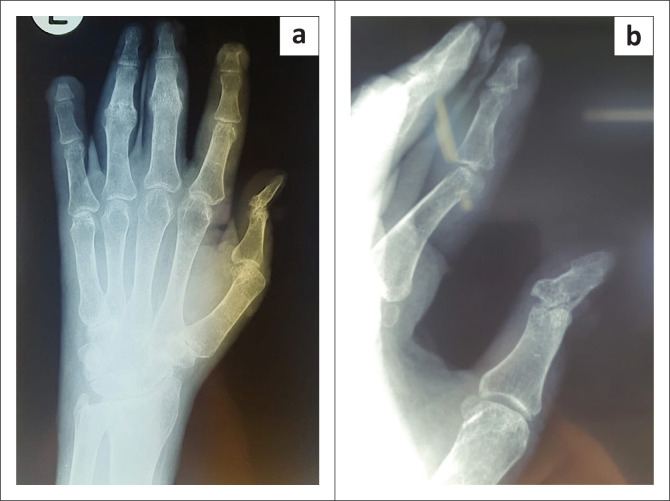
(a and b) X-ray of patient’s left hand, demonstrating osteoarticular destruction at multiple sites.

The patient presented next in July 2021, with worsening skin lesions and progressive deformity of the hands. The left hand was swollen and tender on palpation. There was limited to no range of movement in the fingers of the left hand. The right hand had comparably better range of movement. Hyperkeratinisation of the nails was found on both hands (Figure 1). X-rays of the patient’s hands demonstrated multifocal osteoarticular erosion of joints of the fingers (Figure 2). Skin punch biopsies and nail samples were submitted for laboratory evaluation. Histological examination and mycology cultures were unable to identify any features of fungal infection, and fungal cultures were negative on these specimens.

Despite negative cultures and inconclusive histological findings, a decision was made to admit the patient for a 2-week course of intravenous deoxycholate amphotericin B and oral itraconazole. The patient was subsequently discharged on a continuation of oral itraconazole.

The patient returned to the Infectious Disease clinic two weeks post-discharge, in September 2021 with a remarkable improvement of his skin lesions. The swelling of the right hand was reduced and was no longer tender to palpation. Unfortunately, the fixed deformities found in the hands were unchanged and the patient had a permanent loss of function. The tissue loss of the patient’s nasal alae remained unchanged.

## Discussion

Sporotrichosis most commonly presents as a fixed eruption of pruritic papules and lymphatic spread.^9^ This typical presentation was found at the initial onset of disease in the patient. In its chronic stages, lymphocutaneous sporotrichosis may ulcerate and spread forming hyperkeratotic and hyperpigmented plaques.^10^ The patient displayed these typical plaques as well as pruritic, papular lesions, predominantly found on the hands, arms and face. Erosion and tissue loss of the nasal alae of our patient’s face indicated the advanced nature of his disease. Systemic symptoms are uncommon,^6^ and none were reported by the patient.

Disseminated sporotrichosis can involve the lung, pleura, sinuses, meninges, liver, heart, kidneys, eyes, bone, and joints but remain rare.^3,6,7^ Alcohol consumption, poorly treated or untreated HIV infection, diabetes mellitus, steroid treatment, haematological cancers and solid organ transplant have been identified as major risk factors for the development of disseminated disease.^8,11^ Treatment response and cure rates are substantially lower in immunocompromised patients.^7^ A study by Ramos and colleagues found a cure rate of 84.6% in immunocompetent patients, compared to a cure rate of only 39.3% of immunocompromised patients, emphasising the importance of the immune system in controlling this infection.^7^ The patient had multiple risk factors for the development of disseminated sporotrichosis. In addition, the patient was employed as a gardener, which is an occupational risk factor for infection.

Along with cutaneous involvement, the patient presented with fixed flexion deformities of the ring and middle fingers of both hands with distal interphalangeal joint involvement of the left thumb indicating osteoarticular sporotrichosis, which was confirmed radiologically.

As can be seen in this case, the diagnosis of extra-cutaneous sporotrichosis offers a significant diagnostic challenge.^12^ Microbiological culture remains the gold standard for diagnosis of sporotrichosis.^13^ On direct microscopy, using potassium hydroxide preparations, oval or cigar-shaped fungal structures can be appreciated.^14^ However, microscopy has a low sensitivity and specificity.^13,14^ Histological features, including epidermal hyperplasia, hyperkeratosis, intraepidermal abscesses and mixed granulomas with asteroid bodies can assist in supporting a diagnosis of sporotrichosis but are not specific.^15^ Because of the small number of organisms needed to cause disease, fungal elements are often not seen.^13^ Giemsa-stained smears enhance the sensitivity of microscopy.^14^

For microbiological culture, samples of tissue or fluid from the patient are inoculated onto a Sabouraud dextrose agar plate and incubated at 25°C.^13^ Culture offers better diagnostic accuracy but is often negative because of the low number of organisms present in active disease.^13^ Multiple skin punch biopsies and nail clippings were taken from our patient from 2019 to 2021, offering few diagnostic answers. This highlights the elusive nature of the Sporothrix fungus and difficulties in its diagnosis. Molecular techniques, such as PCR and sequencing, have recently come into practice.^16^ These tests may offer more reliable testing, with higher sensitivities and specificities;^16^ however, these were not yet available at our laboratory.

Itraconazole is the drug of choice for the treatment of sporotrichosis, with a recommended dosing of 200 mg/day for three to six months, depending on the disease severity.^17^ Extended suppressive therapy has been recommended in patients with immunocompromise, to prevent recurrence.^17^ Itraconazole is a fungistatic drug that acts via inhibiting the fungal-mediated synthesis of ergosterol.^14^ It is metabolised by the cytochrome P450 3A4 enzyme; thus drug–drug interactions are an important consideration. Our patient was receiving antiretroviral therapy, therefore close monitoring of adverse events was essential. In particular, interactions between itraconazole and non-nucleotide reverse transcriptase inhibitors, such as efavirenz, are of particular concern with a reduction in itraconazole therapeutic concentrations.^18^ Serum levels of itraconazole or its metabolites have been recommended 14 days after initiation of therapy.^19^ Serum levels above 1 mcg/mL have been associated with treatment success, while levels above 5 mcg/mL have been found to increase risk of toxicity.^19^ Regrettably, such monitoring was not available at our facility.

In severe cases, concomitant use of amphotericin B can be considered, particularly in immunocompromised patients with systemic infection.^14,17^ In this case, an induction phase of 14 days of intravenous deoxycholate amphotericin B, in addition to oral itraconazole was used. Although liposomal amphotericin B is superior to amphotericin B deoxycholate because of a better side effect profile,^20^ it was not available in our facility. On completion of deoxycholate amphotericin B, the patient was discharged on a long course of oral itraconazole. A longer duration of therapy of 6–12 months has been recommended in patients with osteoarticular sporotrichosis.^3,7,14,17^ A month after discharge our patient was reviewed, with a significant improvement in his cutaneous lesions and no further destruction of the involved bones. Our patient was referred to the plastic surgery department and follow-up consultation at our HIV clinic was arranged for the optimisation of adherence to HIV therapy.

## Conclusion

Disseminated sporotrichosis is a potentially debilitating disease. With the difficulties in the diagnosis of Sporotrichosis as described, a high index of suspicion is needed to diagnose and treat timeously. This is essential to avoid possible permanent sequelae of disseminated sporotrichosis, as observed in our case.
